# Prevention of Oxidative Stress-Induced Pancreatic Beta Cell Damage by *Broussonetia kazinoki* Siebold Fruit Extract via the ERK-Nox4 Pathway

**DOI:** 10.3390/antiox9050406

**Published:** 2020-05-10

**Authors:** Hyo-Jin Kim, Donghee Kim, Haelim Yoon, Cheol Soo Choi, Yoon Sin Oh, Hee-Sook Jun

**Affiliations:** 1 College of Pharmacy, Gachon University, Incheon 21936, Korea; hyooojin@gc.gachon.ac.kr; 2 Lee Gil Ya Cancer and Diabetes Institute, Gachon University, Incheon 21999, Korea; dh2388@gachon.ac.kr (D.K.); limtiny@naver.com (H.Y.); cschoi@gachon.ac.kr (C.S.C.); 3 Department of Medicine, College of Medicine, Gachon University, Incheon 21565, Korea; 4 Korea Mouse Metabolic Phenotyping Center, Lee Gil Ya Cancer and Diabetes Institute, Gachon University, Incheon 21999, Korea; 5 Department of Food and Nutrition, Eulji University, Seongnam 13135, Korea; 6 Gachon Medical and Convergence Institute, Gachon Gil Medical Center, Incheon 21565, Korea

**Keywords:** *Broussonetia kazinoki* Siebold fruit extract, diabetes, pancreatic beta cell, oxidative stress, apoptosis

## Abstract

Pancreatic beta cells are vulnerable to oxidative stress, which causes beta cell death and dysfunction in diabetes mellitus. *Broussonetia kazinoki* Siebold (BK) is a widely used herbal medicine, but its potential effects against beta cell death-induced diabetes have not been studied. Therefore, we investigated the protective effect of an ethanolic extract of BK fruit (BKFE) against streptozotocin (STZ)-induced toxicity in pancreatic beta cells. Intraperitoneal injection of STZ in mice induced hyperglycemia; however, oral administration of BKFE significantly decreased the blood glucose level as well as HbA1c levels. BKFE treatment improved glucose tolerance and increased body weight in diabetic mice. Moreover, BKFE treatment resulted in increased serum insulin levels and insulin expression in the pancreas as well as decreased 4-hydroxynonenal levels induced by oxidative stress. Treatment with STZ decreased cell viability of mouse insulinoma cells (MIN6), which was blocked by BKFE pretreatment. BKFE significantly inhibited apoptotic cells and decreased the expression levels of cleaved-caspase-3 and cleaved-poly (ADP-ribose) polymerase (PARP) induced by STZ treatment. Production of reactive oxygen species in STZ-treated MIN6 cells was also significantly decreased by treatment with BKFE. Erk phosphorylation and Nox4 levels increased in STZ-treated MIN6 cells and the pancreas of mice injected with STZ and this increase was inhibited by treatment with BKFE. Inhibition of Erk phosphorylation by treatment with the PD98059 inhibitor or siRNA Erk also blocked the expression of Nox4 induced by STZ treatment. In conclusion, BKFE inhibits Erk phosphorylation, which in turn prevents STZ-induced oxidative stress and beta cell apoptosis. These results suggested that BKFE can be used to prevent or treat beta cell damage in diabetes.

## 1. Introduction

Diabetes is a group of metabolic diseases characterized by hyperglycemia. In general, there are two types of diabetes: type 1 diabetes is caused by the lack of insulin production owing to the destruction of insulin-producing pancreatic beta cells, whereas type 2 diabetes results from insulin resistance in the muscles, liver, and adipose tissues [1,2]. In type 2 diabetes, beta cells fail to compensate for insulin resistance, resulting in the development of hyperglycemia, loss of functional beta cell mass, and finally, insulin deficiency [3]. Therefore, pancreatic beta cell mass is known to play an important role in the development of both types of diabetes [4].

Several factors, such as inflammation, glucotoxicity, lipotoxicity, and glucolipotoxicity, contribute to the progression of diabetes. Persistent hyperglycemia promotes oxidative stress and overproduction of reactive oxygen species (ROS) leading to pancreatic beta cell damage and dysfunction [5]. Oxidative stress is caused by overproduction of ROS or an impaired antioxidant system [6,7]. Pancreatic beta cells are particularly susceptible to oxidative stress and damage due to intrinsically low expression of antioxidant genes [8], which are important factors that lead to apoptosis and a decrease in beta cell mass [2,9,10,11,12].

There is constant search for new drugs from natural products, for herbal medicines that can be used to prevent and treat diabetes [13,14,15,16]. Furthermore, many studies have been performed on natural plant materials that lower blood glucose levels and improve pancreatic beta cell function [17,18,19,20,21]. The “Jeo-sil-ja” in Korea, dried fruits of Japanese paper mulberry (*Broussonetia kazinoki* Siebold, BK), belongs to the Moraceae family and grows in East Asia in countries such as Korea, China, and Japan. It has been used to treat symptoms such as neuralgia, dermatitis, and swelling, and has also been used for its diuretic effects [22]. Furthermore, various reports have detailed the anti-inflammatory, anti-diabetic, and anti-cancer effects of the leaves, twigs, root, and stem barks of BK [23,24,25,26], but very little is known about beneficial effects of BK fruits in diabetes, especially on beta cells. Recently, we reported that an ethanol extract of BK fruit (BKFE) reduced mesangial cell apoptosis induced by palmitate, and this was due to activation of Nrf2 and upregulation of antioxidant genes [27], suggesting that BKFE may have anti-apoptotic effects on beta cells. Moreover, it was reported that stem bark and root bark of BK have anti-apoptotic effects on beta cells [28,29]. Therefore, we hypothesized that BKFE may have antioxidant effects against beta cell death in streptozotocin (STZ)-treated type 1 diabetic mice and MIN6 cells and investigated the signaling pathway involved.

## 2. Materials and Methods

### 2.1. Preparation of BKFE

The powder of dried BK fruits was purchased from an oriental drug store (Kwang Myung Dang Co., Ulsan, Korea), and then an 80% ethanol extract was prepared as described previously [27]. The extract was evaporated in vacuo to obtain a dark brownish residue and dissolved in dimethyl sulfoxide (DMSO) (Duchefa Biochemie B.V., Haarlem, Netherlands) to a concentration of 50 or 400 mg/mL for in vitro or in vivo experiments, respectively.

### 2.2. Animals

Six-week-old male C57BL/6 mice (Orient Bio Inc, Seongnam, Gyeonggi-do, Korea) were maintained in specific-pathogen-free (SPF) animal facilities. All animal experiments were carried out according to a protocol approved by the Institutional Animal Care and Use Committee at Lee Gil Ya Cancer and Diabetes Institute, Gachon University (LCDI-2018-0115). Mice were housed in groups of 3–5 animals/cage under a 12-h light/dark cycle at 23 ± 1 °C, 40–50% humidity. The animals were allowed free access to a standard chow diet and water during the experiment period. After a week of adaptation, mice with similar physical characteristics (i.e., similar age and body weight) were randomly divided (*n* = 7–11) into three groups: Con, vehicle-treated non-diabetic control group; STZ, vehicle and STZ-treated group; and BKFE, BKFE and STZ-treated group. Type 1 diabetes was induced by intraperitoneal (i.p.) injection of 50 mg/kg/day STZ (Sigma, St Louis, MO, USA) freshly prepared in 0.1 M sodium citrate buffer, pH 4.5, for 5 consecutive days. Mice were orally administered BKFE (50 mg/kg/day) or 1.25% DMSO in sterile distilled water containing 2% Tween-80 and 0.5% methylcellulose daily for 8 weeks from 1 day before the STZ injection. Mice with blood glucose levels above 300 mg/dL were considered diabetic. Body weight and blood glucose levels were checked once a week. After 8 weeks, all animals were fasted overnight and sacrificed with the approval of the Institutional Review Board (IRB), and then, pancreatic tissues were collected.

### 2.3. Glucose Tolerance Test (GTT)

A GTT was performed 8 weeks after STZ treatment. Mice were fasted for 18 h and injected i.p. with glucose (2 g/kg). Blood samples were obtained from the tail vein at 0, 15, 30, 60, 90, and 120 min after the glucose injection. Blood glucose levels were measured with a glucose analyzer (OneTouch Ultra, Lifescan, Johnson and Johnson, Milpitas, CA, USA). Each mouse was kept in a separate cage for GTT analysis to minimize stress. Based on the GTT curves, the area under the curve (AUC) was calculated in mg·dL^−1^·min.

### 2.4. Measurement of Serum Insulin Levels

To examine serum insulin levels, mice were fasted for 4 h, and then, blood samples were collected at 8 weeks after BKFE oral intubation. After incubation for 30 min at 25 °C, blood was centrifuged at 3000× *g* for 20 min at 4 °C, and the serum was collected. Insulin levels were quantified using an Ultra Mouse Insulin ELISA kit (Alpco Diagnostics, Windham, NH, USA) according to the manufacturer’s instructions.

### 2.5. Measurement of Hemoglobin A1c (HbA1c) Levels

At 8 weeks after STZ treatment, blood samples were collected from the tail vein of experimental mice. HbA1c levels were measured using a DCA Vantage™ Analyzer (SIEMENS, Tarrytown, NY, USA) and a DCA 2000 Reagent Kit (SIEMENS) following the manufacturer’s instructions. HbA1c levels less than 6% were considered normal [30].

### 2.6. Immunostaining

The pancreas tissues were fixed in a 10% formalin solution for paraffin sectioning. For histological evaluations, these sections were stained with hematoxylin and eosin (H and E). For immunohistochemical analysis, pancreatic sections were dewaxed with xylene and dehydrated with ethanol. After antigen retrieval using 10 mM citric acid buffer (pH 6.0), the sections were permeabilized with 0.2% Triton X-100 in PBS. The sections were blocked in Protein Block Serum-Free Ready-To-Use solution (Dako North America, Inc., Carpinteria, CA, USA) at room temperature for 1 h, and incubated at 4 °C overnight with insulin (ab7842, Abcam, Cambridge, MA, USA), glucagon (G2654, Sigma, St. Louis, MO, USA), 4-hydroxynonenal (4HNE) (ab46545, Abcam), and Nox4 (ab195524, Abcam) antibodies diluted at 1:200 in Antibody Diluent (Dako North America, Inc.). Peroxidase staining was performed with 3,3-DAB (Liquid DAB+ Substrate Chromogen System; Dako North America, Inc.). For immunofluorescence, the samples were incubated with Texas-red (TR) or fluorescein isothiocyanate (FITC)-conjugated secondary antibodies at room temperature for 1 h. The nuclei were stained with 4′,6-diamidino-2-phenylindole (DAPI) (Invitrogen, San Diego, CA, USA) diluted 1:1000, and mounted with fluorescence mounting media (Dako North America, Inc.). These sections were observed using a light microscope or confocal microscope (Carl Zeiss Inc., Oberkochen, Germany). The area of insulin- and glucagon-expressing tissues was calculated using ImageJ software (National Institutes of Health, Bethesda, MD, USA). 

### 2.7. Cell Culture

Mouse insulinoma (MIN6) cells were obtained from American Type Culture Collection (ATCC, Rockville, MD, USA). MIN6 cells were maintained in DMEM containing 10% fetal bovine serum, 100 U/mL penicillin, 100 μg/mL streptomycin, 0.1% β-mercaptoethanol at 37 °C in an atmosphere of 5% CO_2_ in 95% air. In all experiments, the cells were incubated in 10 μg/mL BKFE for 1 h prior to treatment with 2 or 10 mM STZ. MIN6 cells on DMSO media were used as a control. The experiments were performed with cells between passages 27 and 33.

### 2.8. Cell Viability Assay

Cell viability was determined using a CCK-8 assay with the tetrazolium salt WST-8, which produces water-soluble WST-8 formazan. Briefly, MIN6 cells (2 × 10^4^ cells/well) were seeded in 96-well plates and incubated overnight at 37 °C. The cells were pretreated with BKFE for 1 h and then incubated with or without STZ for 24 h at 37 °C. Viable cells were measured by using D-Plus™ CCK cell viability assay kit (Dongin LS, Seoul, Korea). After incubation for 3 h, the absorbance of each sample at a wavelength of 450 nm was detected using a VersaMax Microplate Reader (Molecular Devices, LLC, Sunnyvale, CA, USA).

### 2.9. Annexin V and Propidium Iodide Staining

Apoptotic cells were detected using an Annexin V FITC/propidium iodide (PI) apoptosis detection kit (BD Biosciences, Franklin Lakes, NJ, USA) according to the manufacturer’s instruction. In brief, the cells were trypsinized and collected together with floating dead cells. The cells were resuspended in a 1× binding buffer. The cells were stained with annexin V-FITC and PI for 15 min at room temperature in the dark. Then, the cells were analyzed using flow cytometry (FACS LSRⅡ, BD Biosciences, CA, USA). Annexin V^+^/PI^−^ population defined early apoptotic cells, and annexin V^+^/PI^+^ population defined late apoptotic cells. Apoptotic cells were calculated by the sum of early and late apoptotic cells and expressed as a percentage of the total number of cells.

### 2.10. Measurement of ROS Levels

MIN6 cells were incubated with 5 μM of 2,7-dichlorodihydrofluorescein diacetate (DCFH-DA, Invitrogen) for 15 min at 37 °C. The cells were then harvested and fixed with 8% neutral buffered formalin (NBF, Sigma, St. Louis, MO, USA) for 20 min on ice. The cells were resuspended in DPBS, and the resulting fluorescent compounds (DCF) were measured by FACS LSRⅡ using the CellQuest™ Pro Software (BD Biosciences).

### 2.11. Western Blotting

The cells were harvested and lysed using a lysis buffer (mammalian protein extraction reagent; Thermo, Bremen, Germany) with a mixture of protease and phosphatase inhibitor cocktail. Total proteins were quantified using the BCA assay, separated electrophoretically, and transferred onto polyvinylidene difluoride membranes (PVDF, Millipore, Billerica, MA, USA). The membranes were blocked with 5% skimmed milk at room temperature for 1 h, and then incubated with primary antibodies (5% skimmed milk with 1:2000) against anti-caspase-3 (9662S, Cell Signaling Technology, Boston, MA, USA), anti-PARP (9542, Cell Signaling Technology), anti-NADPH oxidase (Nox)4 (ab195524, Abcam), anti-p-Erk (p-p44/42 MAPK) (9101S, Cell signaling Technology), and anti-Erk (9102S, Cell signaling Technology) at 4 °C overnight. After washing with TBST, the membranes were incubated with horseradish peroxidase-conjugated secondary antibodies (Santa Cruz Biotechnology; Jackson Immunoresearch, West Grove, PA, USA) for 1 h at room temperature. The blots were developed using Luminata™ Forte Western HRP Substrate (Millipore) and visualized using a LAS-4000 mini system (Fujifilm Corp., Tokyo, Japan). The intensity of protein bands was quantified using the ImageJ software (National Institute of Health).

### 2.12. Quantitative Real Time (RT)-Polymerase Chain Reaction (PCR)

Total RNA was extracted from the cultured cells using TRIZOL reagent (Invitrogen) and cDNA was synthesized using a PrimeScript 1st strand cDNA synthesis kit (TaKaRa Bio Inc., Shiga, Japan). Quantitative RT-PCR was performed using SYBR Master Mix (TaKaRa Bio Inc.) and a CFX384 real-time PCR system (BioRad, Hercules, CA, USA) to detect mRNA expression levels. Nox4 primer sequences were as follows: 5′-CCACAGACCTGGATTTGGAT-3′ and 5′-TGGTGACAGGTTTGTTGCTC-3. Cyclophilin was used as an internal control, with the following primers: 5′-TGGAGAGCACCAAGACAGACA-3′ and 5′-TGCCGGAGTCGACAATGAT-3′. PCR was carried out for 40 cycles (10 min at 95 °C, and 40 cycles of 10 s at 95 °C and 1 min at 60 °C). The relative copy number was calculated using the threshold crossing point (Ct) based on the 2-ΔΔCt calculations.

### 2.13. Transfection

MIN6 cells (5 × 10^5^ cells/60 mm dish) were plated and transfected with 20 nM siRNA for Erk2 (p42 Mapk1; siErk) or scrambled siRNA (siCon) (Bioneer, Daejeon, Korea) using the Lipofectamine RNAiMAX (Invitrogen) reagent according to the manufacturer’s instructions. At 24 h after transfection, the medium was replaced with a complete culture medium containing DMSO or BKFE, incubated for 1 h, and then the cells were treated with STZ for various time periods. Erk2 siRNAs were transfected into the cells by mixing of siRNA#1 and siRNA#2. The sequences for siErk2 were as follows: siRNA#1, GACAUGGAGUUGGACGAC and siRNA#2, AGUCGUCCAACUCCAUGUC.

### 2.14. Statistical Analysis

Statistical analysis was estimated by one-way analysis of variance (ANOVA) with Tukey’s multiple comparison test using the GraphPad Prism version 5 (GraphPad software Inc., San Diego, CA, USA). All data are expressed as mean ± standard error of the mean (SEM). The value of statistical significance was set at *P* < 0.05.

## 3. Results

### 3.1. BKFE Ameliorates STZ-Induced Diabetes

**Figure 1:**
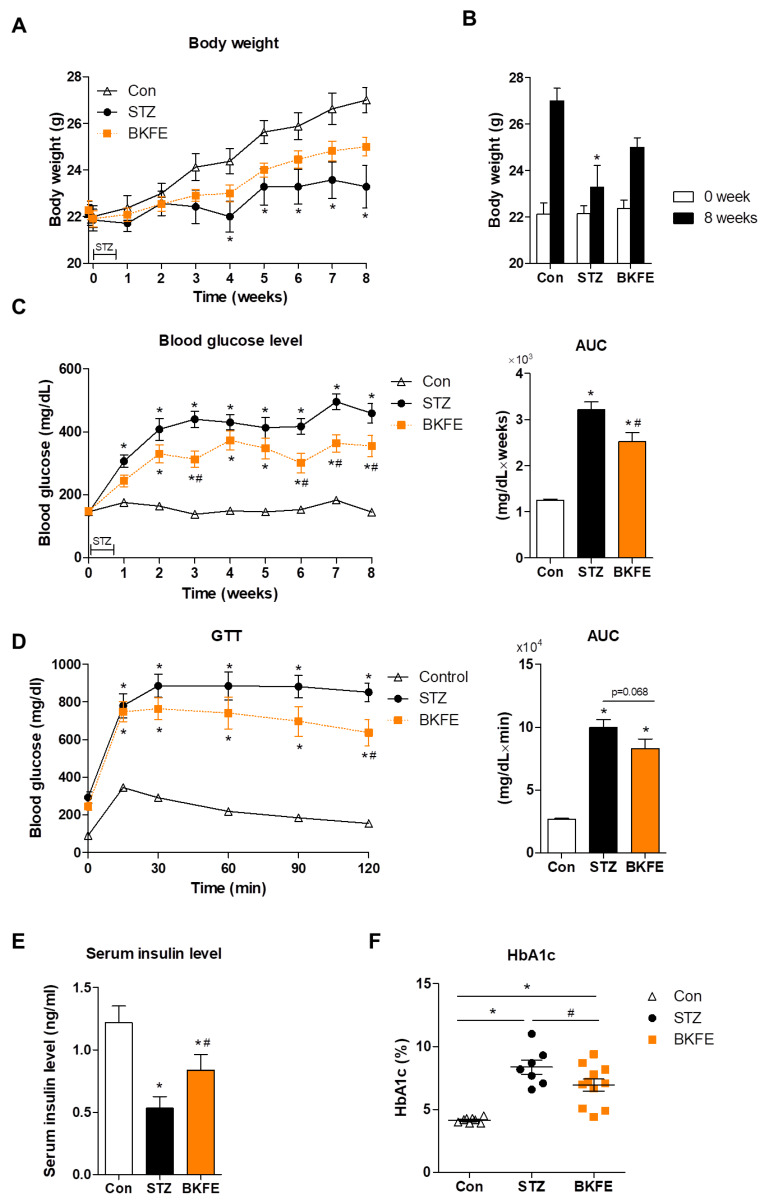
BKFE ameliorates STZ-induced diabetes. Diabetes was induced by multiple (for five consecutive days) low-dose (50 mg/kg) intraperitoneal (i.p.) injections of streptozotocin (STZ) in C57BL/6 mice. Mice were orally administered either vehicle or ethanolic extract of *Broussonetia kazinoki* Siebold fruit (BKFE) (50 mg/kg) for eight weeks from one day before STZ injection and then sacrificed. Non-diabetic mice were administered with vehicle as a control. (**A**) Changes in body weight, (**B**) body weight before and after treatment for eight weeks, and (**C**) blood glucose levels for eight weeks were measured, and then the area under the curve (AUC) was calculated in mg·dL^−1^·week based on the blood glucose levels. (**D**) Glucose tolerance test (GTT) was performed and AUC was calculated in mg·dL^−1^·min based on the GTT curves. (**E**) Insulin and (**F**) HbA1c were measured after eight weeks of treatment. Data are presented as mean ± SEM (*n* = 7–11). * *p* <0.05 vs. Con; # *p* <0.05 vs. STZ; Con, Vehicle-treated nondiabetes; STZ, Vehicle + STZ; BKFE, BKFE + STZ.

To examine the beneficial effects of BKFE in diabetes, we administered BKFE (50 mg/kg/day) for eight weeks to STZ-injected mice. While the body weight was significantly reduced from four weeks after STZ injection compared with that of control mice, the body weight loss was restored with BKFE treatment (Figure 1A). After eight weeks of treatment, body weight was higher in the BKFE-treated group than in the STZ-injected mice treated with vehicle (Figure 1B). We observed that STZ treatment induced an increase in blood glucose levels, however, BKFE administration significantly reduced the blood glucose levels compared with the STZ-alone group (Figure 1C). The AUC indicated a significant increase in the STZ-treated group compared with the control group, and it was significantly decreased by the BKFE treatment. To assess the effect of BKFE administration on glucose regulation, we performed i.p. GTT in STZ-induced diabetic mice with or without BKFE at 8 weeks. During the GTT, blood glucose level peaked at 15 and 30 min after the glucose injection, and remained significantly increased at all times in the diabetic groups compared with the control group. When compared with the STZ-alone group, BKFE treatment lowered blood glucose levels at 30 min, with a significant decrease being observed at 120 min after the glucose injection. The AUC graph showed that STZ-injected mice showed impaired glucose tolerance, which was reduced with BKFE treatment (*p* = 0.068). The control mice showed a normal clearance of exogenous glucose (Figure 1D). STZ-treated diabetic mice showed a decrease in insulin level as well as an increase in HbA1c level compared with the control mice, and these levels were reversed in the BKFE-treated diabetic mice group by the end of the experimental periods (Figure 1E,F).

### 3.2. BKFE Reduces Beta Cell Damage in STZ-Induced Diabetic Mice

**Figure 2:**
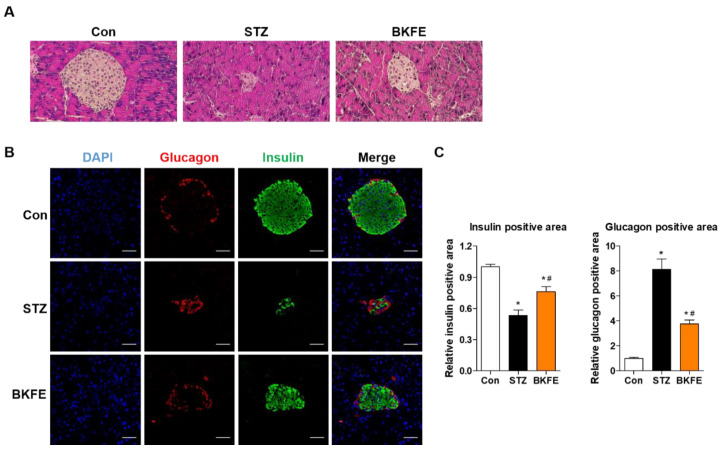
BKFE reduces beta cell damage in STZ-induced diabetic mice. Histological analysis of pancreas sections. (**A**) Representative images showing the islets by hematoxylin and eosin (H and E) staining (original magnification, 200×). (**B**) Representative images showing the expression of insulin (green) and glucagon (red) by immunofluorescence staining. The nuclei were counterstained with 4′,6-diamidino-2-phenylindole (DAPI) (blue). (Scale bars, 50 μm). (**C**) Relative insulin- and glucagon-positive areas were quantified using the ImageJ software. Data are presented as mean ± SEM (*n* = 5). * *p* <0.05 vs. Con; # *p* <0.05 vs. STZ; Con, Vehicle-treated non-diabetes; STZ, Vehicle + STZ; BKFE, BKFE + STZ.

To evaluate changes in the beta cell area in BKFE-treated mice, we measured insulin-positive cells by staining pancreatic sections from each group with an anti-insulin antibody. As shown in Figure 2, the islets from control mice had a regular morphology with beta cells located in the center, but these were smaller and destroyed in the STZ-induced diabetic mice. However, BKFE treatment changed the histopathological phenotype by restoring islet mass (Figure 2A,B). Quantification of insulin-positive cells showed that STZ treatment significantly decreased the mass of beta cells, which was reversed by the BKFE treatment (Figure 2C). We also evaluated the composition of non-beta cells in the islets and found that glucagon-secreting alpha cells in control mice were located in the periphery. After STZ administration, the distribution of alpha cells became more centrally localized, but this changed with the BKFE treatment (Figure 2B). Quantitative analysis showed that the glucagon-positive area was increased in STZ-injected mice, and it was significantly decreased by treatment with BKFE (Figure 2C).

### 3.3. BKFE Reduces Oxidative Stress in Pancreatic Islets of STZ-Induced Diabetic Mice

**Figure 3:**
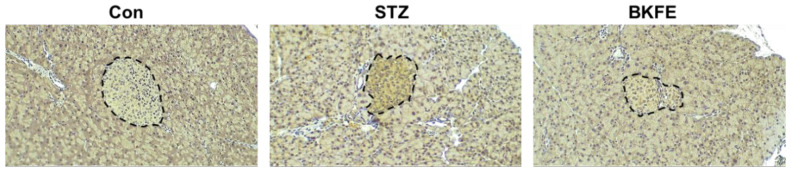
BKFE reduces oxidative stress in pancreatic islets of STZ-induced diabetic mice. Immunohistochemical detection of 4-hydroxynonenal (4HNE) as a marker for oxidative stress was performed in pancreas sections from experimental mice treated with STZ and/or BKFE and non-diabetic controls. 4HNE staining is shown as a brown reaction area demarcated by the black dotted line, and the nuclei are stained with hematoxylin. Representative microscopy images were examined by light microscopy (original magnification, 100×, *n* = 4). Con, Vehicle-treated nondiabetes; STZ, Vehicle + STZ; BKFE, BKFE + STZ.

STZ is known to induce ROS production and increase cytotoxicity in pancreatic beta cells [31]. Therefore, we investigated the changes in ROS generation in islets from STZ- and BKFE-treated mice by immunostaining with an antibody against 4HNE, which is a marker of ROS production [32,33]. As shown in Figure 3, 4HNE expression was not found in control mice, but its levels were increased in the islets from STZ-injected mice. However, BKFE treatment significantly attenuated STZ-induced ROS production (Figure 3).

### 3.4. BKFE Protects MIN6 Cells From STZ-Induced Toxicity 

**Figure 4:**
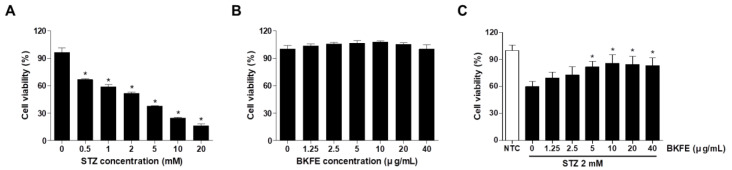
BKFE protects MIN6 cells from STZ-induced toxicity. (**A**) MIN6 cells were treated with various concentrations of STZ for 24 h. (**B**) Cells were treated with various concentrations of BKFE for 24 h. (**C**) Cells were pretreated with the indicated concentrations of BKFE for 1 h, and then incubated with 2 mM STZ for 24 h. A CCK-8 assay was performed to determine the cell viability. Data are presented as mean ± SEM from three independent experiments. * *p* <0.05 vs. 0 (non-treated cells); NTC, Negative control.

As we observed that the reduced number of insulin-positive cells in islets from STZ-induced diabetic mice had increased with BKFE treatment, we wanted to confirm this effect in MIN6 cells, a mouse beta cell line. MIN6 cells were treated with various concentrations (0.5–20 mM) of STZ for 24 h, and then, cell viability was measured. As shown in Figure 4A, cell viability was decreased in a dose-dependent manner compared with the control. Treatment of MIN6 cells with 1.25–40 μg/mL BKFE for 24 h was not toxic (Figure 4B). To investigate the effects of BKFE on STZ-induced cell death, the cells were pretreated with various concentrations of BKFE for 1 h and then with 2 mM STZ for 24 h. Cell viability was decreased on treatment with 2 mM STZ compared with control cells, and pretreatment with BKFE increased the cell viability dose-dependently, peaking at 10 μg/mL of BKFE. Further increase in cell viability was not observed after treatment with 20 or 40 μg/mL BKFE (Figure 4C).

### 3.5. BKFE Inhibits STZ-Induced Apoptosis in MIN6 Cells

**Figure 5:**
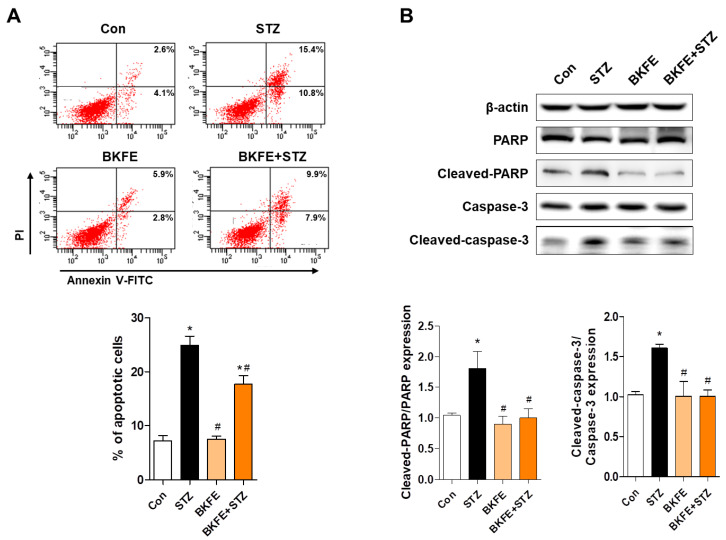
BKFE inhibits STZ-induced apoptosis in MIN6 cells. Cells were treated as described in Figure 4C, and (**A**) apoptotic cells were labeled using annexin V-FITC/PI and detected with flow cytometry. Percentage on representative dot plots indicates the early (the lower right quadrant) and late (the upper right quadrant) apoptotic cells. Graph shows quantitative data for the percentage of the sum of early and late apoptotic cells. (**B**) Expression levels of cleaved-PARP and cleaved-caspase-3 were detected by Western blotting. Densitometry quantification of the relative expression of proteins was performed using the ImageJ software. Data are presented as mean ± SEM from three independent experiments. * *p* <0.05 vs. Con; # *p* <0.05 vs. STZ; Con, Vehicle + citrate buffer; STZ, Vehicle + STZ; BKFE, BKFE + citrate buffer; BKFE + STZ, BKFE + STZ.

To determine the anti-apoptotic effect of BKFE, we performed annexin V-FITC and PI staining after STZ treatment with or without BKFE treatment. As shown in Figure 5A, the proportion of annexin V FITC^+^/PI^−^ cells was increased with STZ treatment, and treatment with 10 μg/mL BKFE significantly inhibited this increase (Figure 5A). Western blot analysis of apoptosis-related proteins showed that STZ treatment increased the expression levels of proapoptotic proteins such as cleaved-caspase-3 and cleaved-PARP, whereas treatment with BKFE markedly reduced the expression of proapoptotic proteins. Quantification results showed that the expression ratio of cleaved-PARP/PARP and cleaved-caspase-3/caspase-3 was significantly increased in STZ-treated cells compared with control cells, and these were inhibited after STZ treatment with BKFE (Figure 5B).

### 3.6. BKFE Inhibits STZ-Induced Production of ROS by Downregulation of P-Erk/Nox4 Pathway in MIN6 Cells and Mice

**Figure 6:**
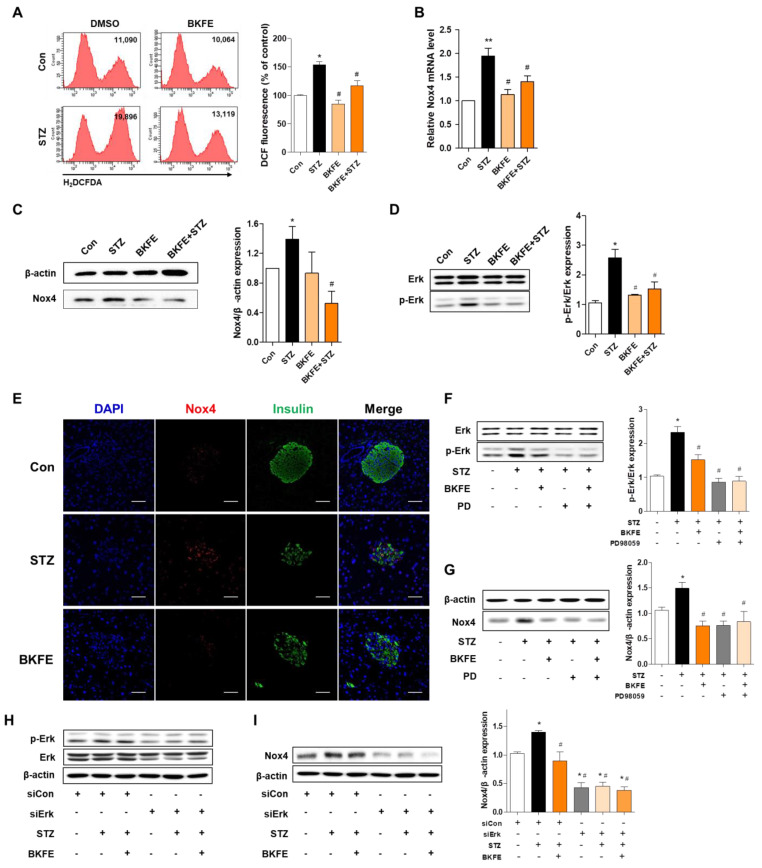
BKFE inhibits STZ-induced production of ROS by downregulation of p-Erk/Nox4 pathway in MIN6 cells and mice. (**A**) The cells were treated as described in Figure 5, and the cells were stained with H_2_DCFDA and analyzed by flow cytometry. Values on the representative flow cytometry data indicate the DCF fluorescence intensity of whole cells. The bar graph shows quantitative data for the percentage of intracellular ROS generation. (**B**) Nox4 mRNA values were analyzed by quantitative RT-PCR from three independent experiments. MIN6 cells were pretreated with 10 μg/mL of BKFE for 1 h and then further incubated with 10 mM STZ for 1 h (**C**) or 30 min (**D**) to detect Nox4, Erk, and p-Erk levels. Con, Vehicle + citrate buffer; STZ, Vehicle + STZ; BKFE, BKFE + citrate buffer; BKFE + STZ, BKFE + STZ. (**E**) Immunohistochemical detection of Nox4 in pancreatic sections from experimental mice treated with STZ and/or BKFE and non-diabetic controls. Representative images showing the expression of Nox4 (red) and insulin (green) by immunofluorescence staining. The nuclei were counterstained with DAPI (blue). (Scale bars, 50 μm). Con, Vehicle + citrate buffer; STZ, Vehicle + STZ; BKFE, BKFE + STZ. (**F**–**G**) MIN6 cells were pretreated with 10 μg/mL of BKFE for 1 h in the absence or presence of PD98059 (10 μM for 2 h), an Erk inhibitor, followed by 10 mM STZ treatment for 30 min to detect Erk and p-Erk (**F**) or 1 h to detect Nox4 levels (**G)**. MIN6 cells were pretreated with 10 μg/mL BKFE for 1 h in the absence or presence of Erk siRNA (20 nM for 24 h), followed by treatment with 10 mM STZ for 30 min to detect Erk and p-Erk (**H**) or 1 h to detect Nox4 levels **(I**). The relative expression levels of Nox4 (C, G and I) and p-Erk (D and F) were normalized to that of β-actin or total-Erk and quantified using the ImageJ software. (F-I) +, Present; −, Absent. Data are presented as mean ± SEM from three independent experiments. * *p* < 0.05, ** *p* < 0.01 vs. Con or siCon only; # *p* < 0.05 vs. STZ or siCon + STZ.

As we found that ROS production in STZ-injected mice was reduced with BKFE treatment, we endeavored to confirm this effect using MIN6 cells, and investigated the signaling pathway involved in ROS production. ROS generation was measured with DCF fluorescence intensity after STZ treatment with or without BKFE treatment. Exposure to STZ-alone induced a significant increase in DCF fluorescence, and this increase was prevented by pretreatment with 10 μg/mL BKFE (Figure 6A). To determine whether the inhibitory effect of BKFE on STZ-induced oxidative stress was associated with the reduction of an oxidant gene, we measured mRNA expression levels of Nox4 using quantitative RT-PCR. A 1.95-fold increase in Nox4 mRNA after STZ treatment was observed, which was significantly prevented by BKFE pretreatment (1.4-fold of basal level) (Figure 6B). Protein levels of Nox4 were also increased one hour after STZ stimulation (1.35-fold) compared with that in control cells, and were significantly reduced by pretreatment with 10 μg/mL of BKFE (0.5-fold of basal level) (Figure 6C). The Erk pathway has been shown to be involved in the induction of Nox4 [34]; therefore, we examined the phosphorylation of Erk in the absence or presence of BKFE. As shown in Figure 6D, p-Erk was increased in STZ-treated cells, and this activation was significantly decreased with BKFE pretreatment (Figure 6D). The p-Erk/Erk ratio significantly increased by 2.56-fold after STZ treatment, which decreased with BKFE pretreatment (1.5-fold of basal level). In addition, higher levels of Nox4 protein was detected in the pancreas of STZ-injected mice compared to those of control mice; in contrast, BKFE treatment reduced the levels of this protein (Figure 6E), which is consistent with the findings for MIN6 cells. Next, to examine whether p-Erk regulates Nox4 expression, MIN6 cells were treated with PD98059 (p-Erk inhibitor) or ERK siRNA (20 nM) and then treated with BKFE and STZ. We found that PD98059 pretreatment significantly inhibited Erk phosphorylation and also blocked the expression of Nox4 induced by STZ treatment (Figure 6F,G). Additive or synergistic effects of p-Erk blockade and inhibition of Nox4 expression by BKFE and PD98059 cotreatment were not observed (Figure 6F,G). Erk2 phosphorylation was significantly reduced 24 h after Erk siRNA transfection (Figure 6H); STZ-induced p-Erk and Nox4 upregulation was inhibited in Erk siRNA transfected cells compared with scrambled siRNA transfected cells (Figure 6H,I). These results suggest that the antioxidant effect of BKFE is caused by down-regulation of Nox4 and ROS production due to inhibition of Erk phosphorylation.

## 4. Discussion

Hyperglycemia in diabetes contributes to the generation of ROS, which mediates beta cell damage and loss of beta cell mass [5,35,36]. Since pancreatic beta cells are vulnerable to oxidative stress due to low expression of antioxidant genes such as superoxide dismutase and catalase [8], the prevention of ROS production is an important therapeutic strategy for diabetes.

BK has been used as a traditional medicine, and its different parts have been reported to have various pharmacological activities. In particular, stem bark from BK shows anti-hyperglycemic activity by inducing insulin levels in diabetic Otsuka Long-Evans Tokushima fatty (OLETF) rats [23] and inhibiting cytokine-induced beta cell apoptosis in RINm5F cells [29]. Recently, we found that the ethanol extract of fruits have protective effects against lipotoxicity in mesangial cells [27]. In this study, we investigated the potential antioxidant effects of BKFE on STZ-induced beta cell damage both in vivo and in vitro.

We confirmed that STZ injection induced type 1 diabetic features such as hyperglycemia, loss of body weight, decrease in insulin level, increase in HbA1c level, and glucose intolerance, as previously reported [37]. However, these symptoms were markedly reduced in diabetic mice treated with BKFE. Examination of the cellular content of islets after STZ treatment indicated a reduction in the proportion of insulin-positive cells compared with that in normal controls, which is consistent with previous reports [38]. However, we found that BKFE administration improved beta cell function and beta cell number in STZ-treated mice. These results suggested that BKFE exerts an anti-diabetic effect by improving insulin secretion by restoring islet mass in STZ-induced diabetic mice.

STZ is a widely-used chemical that induces experimental diabetes in animals and can induce cells to produce several types of ROS, including superoxide anion, hydroxyl radical, and H_2_O_2_ [31]. In addition, STZ induces DNA damage, which leads to activation of PARP, which then initiates the programmed cell death pathway [31,39]. In the present study, exposure of islet or MIN6 cells to STZ significantly increased intracellular ROS production, which was accompanied by a marked decrease in cell viability, augmentation of PARP/Caspase-3 activity, and increase in apoptosis. However, BKFE treatment significantly reduced the expression of biomarkers of oxidative stress and apoptosis in islets and MIN6 cells. BK root bark has also been reported to possess potent antioxidant activity via suppression of nitric oxide (NO) and H_2_O_2_ production in cytokine-treated RINm5F cells [29]. These results suggested that the amelioration of ROS production is one of the important effects in the amelioration of STZ-induced islet apoptosis and diabetic symptoms.

Nox plays a major role in ROS production by promoting the transfer of electrons via the Nox catalytic subunit [40,41]. Seven Nox isoforms (Nox1-5, DUOX 1/2) with different physiological functions have been identified [42]. Among the various isoforms of Nox, pancreatic beta cells express Nox1, Nox2, and Nox4, and these Nox isoforms can positively and negatively regulate insulin secretion and cell survival [43]. Nox4, as a major source of ROS production in beta cells, has been shown to be involved in the pathogenesis of diabetes. Increased expression of Nox4 in the kidney from diabetic mice/rat has been observed, and STZ-treated MIN6 cells also have upregulated Nox4 mRNA expression [44,45]. Wang X et al. demonstrated that Nox4 inhibitors conferred protective effects against cytokines and glucolipotoxicity-induced beta cell damage [46].

Nox4 expression has been reported to be upregulated by various stimuli such as ER stress, shear stress, and hypoxia, and the PKC, AMPK, Smad, and Erk signaling pathway is involved in the regulation of Nox4 expression [34,47,48,49,50]. To investigate the mechanisms involved in the regulation of Nox4 expression by STZ and BKFE treatment, we determined the expression levels of p-AMPKα, p-PKC, and p-Erk, and found that only p-Erk was regulated by STZ and BKFE treatment (data not shown). The levels of p-AMPK and p-ERK were decreased and increased, respectively, by STZ treatment, but that of p-PKC did not change. BKFE pretreatment did not affect the STZ-induced decrease of p-AMPK, however the increase in p-Erk by STZ was inhibited by BKFE pretreatment in MIN6 cells. Horiuchi Y et al. reported that the Erk pathway plays an important role in regulating cell proliferation, differentiation, and apoptosis [51]. In addition, reduction in Erk pathway signaling has been reported to inhibit beta cell apoptosis [52], and specific blockade of the Erk pathway improves insulin resistance in diabetic mice [53]. Previous reports including our results suggested that the activation of Erk is a critical step in the regulation of STZ- or angiotensin II-induced Nox4 expression [54,55]. Some potential antioxidant agents, such as hispidin and sodium hydrogen sulfide, markedly reduced p-Erk, and treatment with an Erk inhibitor (U0126) reduced high glucose-induced cardiomyocyte injury by decreasing ROS generation [56]. These results indicated that the p-Erk/Nox4 pathway might be involved in the regulation of beta cell apoptosis through a decrease of STZ-derived ROS production.

BK has bioactive phytochemicals such as isoprenylated/prenylated flavan, diprenylated flavonols, and flavonoids alkaloids [57,58,59]. Kanizol B, kanizol C, kanizol U, and isokanizol D from root bark have been previously identified, and showed physiological effects such as anti-apoptotic, anti-hyperglycemic, anti-inflammatory, and anti-diabetic effects [28,29,57,60]. The specific bioactive phytochemical producing the antioxidant effect in BKFE was not verified in this study. Flavonoids from fruits might be involved, and further studies for quantification and/or identification of flavonoids in BKFE are required.

## 5. Conclusions

p-Erk-mediated Nox4 upregulation is one of the mechanisms of ROS generation in STZ-treated beta cells. Our results suggest that BKFE protects beta cells against oxidative stress and apoptosis through the deactivation of Erk. The identification of ROS-induced apoptosis and related signaling modulated by BFKE is an important step for the development of targeted approaches to reduce beta cell oxidative stress in diabetes.
